# The influence of climate change on children attending primary care in Isiolo County, Northern Kenya

**DOI:** 10.4102/phcfm.v18i1.5259

**Published:** 2026-01-27

**Authors:** Beatrice W. Muhu, Christian L. Lokotola, Robert Mash

**Affiliations:** 1 Division of Family Medicine and Primary Care, Faculty of Medicine and Health Sciences, Stellenbosch University, Cape Town, South Africa

**Keywords:** climate change, planetary health, child health, primary care, primary health care, Kenya, mixed methods

## Abstract

**Background:**

Climate change has an adverse impact on health in Eastern Africa. Climate-sensitive diseases pose a threat to the health, growth and development of children.

**Aim:**

To determine the influence of climate change on children attending primary care in Isiolo County, Northern Kenya.

**Setting:**

The study was undertaken in Isiolo County Referral Hospital in Isiolo County, Northern Kenya.

**Methods:**

Convergent mixed methods research design. Quantitative data on climate variability and disease patterns were collected over the last 5 years and analysed using the Statistical Package for Social Sciences. Qualitative data from 12 interviews of parents with children under 5 years and six interviews of healthcare workers were analysed with Atlas-ti using the framework method.

**Results:**

The county experienced climate fluctuation between 2019 and 2023, characterised by reduced rainfall, high temperatures, food insecurity, reduced access to water and flash floods. Families were vulnerable to the effects of these climate shocks because of limited finances. Primary care services were of low quality and lacked resilience. Healthcare workers reported limited medical resources, healthcare worker shortages and overcrowding in hospitals. Health effects reported by parents included malaria, pneumonia, diarrhoeal diseases and mental health illnesses. Social effects reported were displacement, child neglect and disruption to education.

**Conclusion:**

Climate change has had a substantial impact on children’s health and social circumstances. Families that are dependent on public sector health services are vulnerable and lack the resilience needed to cope with climate stressors. The health facilities also lack the resilience needed to respond adequately to the challenges of climate change.

**Contribution:**

This study will strengthen climate and health data and improve policies to address regional community needs. It also demonstrates that improving healthcare financing will impact healthcare system resilience.

## Introduction

Climate change is part of an ecological crisis that has significant health and social effects.^1^ The African continent, especially sub-Saharan Africa, is particularly vulnerable to climate change.^2^ Various countries have experienced unprecedented weather conditions, such as Cyclone Idai in Zimbabwe,^3^ severe flooding in north-eastern Nigeria,^4^ and heat stress amidst poor rainfall in many other arid regions.^5^ The north, west and coastal regions of Kenya have witnessed significant climate changes.^6^ Isiolo County, which is situated in the Arid and Semi-Arid Lands (ASAL) of Kenya, has been experiencing a gradual reduction in rainfall regularity and intensity.^7^ Temperatures have also been steadily rising with a severe drought.^8^ This has compromised food security and water resources, as well as natural ecosystems.^9^ Pastoralism and agro-pastoralism, being the primary forms of livelihood, have been adversely affected by these climatic changes. Sparse crop production and limited pastures have led to an upsurge in malnutrition and other climate-sensitive diseases in the region.^10^

Children, particularly under 5 years, are among the most vulnerable.^11^ They often bear a larger burden of disease because of climate change.^4^ While Kenya has made remarkable progress in reducing under-5 mortality rates in the last decades, there still exist challenges such as climate change that can undermine these efforts.^12^

One of the main diseases is malnutrition. Globally, and particularly in sub-Saharan Africa, acute or chronic malnutrition is a major cause of death among children.^13^ Furthermore, malnutrition directly increases the risks of infections such as malaria, respiratory infections, meningitis and acute febrile illnesses.^14^ Gastroenteritis and other waterborne diseases are also more common because of poor water quality and quantity with increased death and disability.^5^ Consequently, the long-term growth, development and well-being of children under 5 years of age are at risk as a result of climate change.^15^

Childhood illnesses and deaths negatively impact society’s psychosocial health and economic productivity.^16^ Parents to sick children experience higher levels of anxiety and depression as well as increased cardiovascular disease risk.^17^ In regions with poor access to primary care services, families tend to struggle with poverty because of increased out-of-pocket healthcare expenses related to childhood diseases.^18^

Primary care workers, responsible for the provision of health care services to children in this region, are often unable to effectively provide quality healthcare because of several factors.^19^ These include limited infrastructure, lack of basic equipment, medications and ambulance services. They also face their own mental health challenges, including anxiety or depression, because of a lack of support, isolation and excessive stress.^18^

This study is important as it investigates the influence of climate change on children in primary care in this region. While climate change has been linked to adverse health outcomes in children, making direct cause-and-effect connections is complex and challenging.^20^ The study may motivate policymakers to formulate and implement adaptation responses to the drought in Isiolo County. Primary care workers will also gain knowledge of the nexus between the diseases they manage and climate change. Few scientific studies have been conducted locally to evaluate the impact of climate change, especially its effects on health and healthcare. This study will add to growing research on the relationship between climate change and health in sub-Saharan Africa, especially in Kenya. It will also encourage further research in other regions affected by climate hazards, such as flooding in Western Kenya and rising water levels in the Rift Valley lakes.

## Research methods and design

### Study design

A convergent parallel mixed methods design was used to collect both quantitative and qualitative data.

### Conceptual framework

**FIGURE 1:**
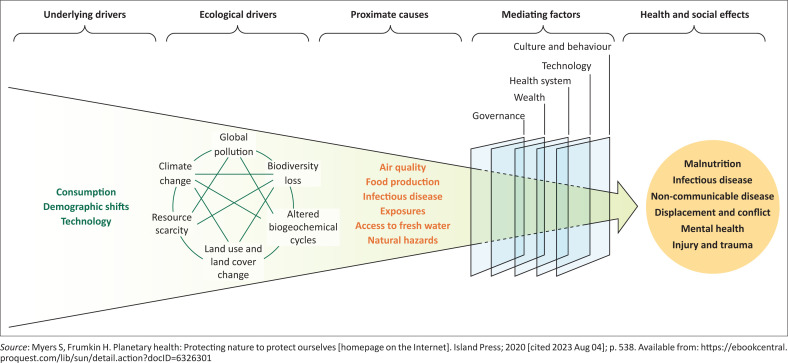
Conceptual framework.

Figure 1^21^ shows the conceptual framework for the study. Climate change is seen as one ecological driver of the current crisis. These drivers have health and social effects via several pathways, such as air quality, food production, infectious disease exposures, access to fresh water and natural hazards, such as drought and extreme temperatures. These proximate causes lead to a wide variety of health effects, including malnutrition, infectious diseases, non-communicable diseases, mental health disorders, injury and trauma. These can be compounded by social effects such as conflict and displacement. The effects are mediated by factors such as the strength of the health system, wealth of the population, effective governance, available technology as well as local culture and belief systems.

### Study setting

**FIGURE 2:**
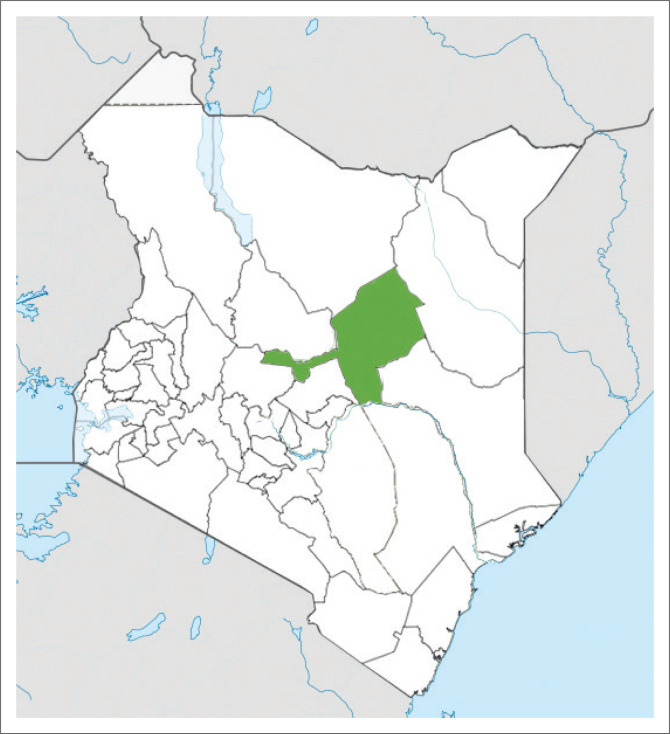
A map of Kenya showing the location of Isiolo County.

The study took place in Isiolo County, which covers approximately 25 700 square kilometres. It is located in the Upper North Eastern Region of Kenya and has six sub-counties and two constituencies (see Figure 2). The population of the county is approximately 268 002 with a population density of 11 persons per square kilometre. The ethnic communities are mainly Maasai, Borana, Somali, Meru and Turkana. Their main economic activities include pastoralism, subsistence farming and small-scale trading within the urban centres. The study was mainly undertaken in Isiolo County Referral Hospital in Isiolo Town and Isiolo sub-county. This is one of the main government health facilities with a 154-bed capacity. It offers both primary and secondary healthcare including paediatric and child health services. Climate change has adversely affected healthcare service delivery; however, health facility preparedness and climate response programmes are inadequate. The hospital serves a population of approximately 121 000 who reside in both rural and urban areas of the county.

### Quantitative data

#### Data sources

Quantitative data on climate variability patterns and related parameters such as temperature, rainfall, vegetation index, distance to water sources and food insecurity were collected from the National Drought Management Authority,^22^ and global weather databases (Table 1).^23^ Morbidity patterns of climate-sensitive diseases such as malaria, diarrhoeal diseases, respiratory tract infections and malnutrition were collected from the Ministry of Health and Health Management Information Systems in Isiolo County.^24^

**TABLE 1:** Quantitative data sources.

Source	Climate exposures and climate-sensitive diseases
	**Climate parameters**
National Drought Management Authority	Rainfall
	Vegetation index
	Distance to water
	Food insecurity
Internet Sources	Temperature
	**Climate-sensitive diseases**
Isiolo County Health Management Information Systems	Malaria
	Upper respiratory tract infections
	Pneumonia
	Diarrhoeal diseases
Ministry of Health	Malnutrition

#### Data analysis

Quantitative data were captured in Microsoft^®^ Excel. The vegetation index (VI) is a single value that quantifies the amount of vegetation in a remote sensing image. It is calculated by combining multiple spectral bands to enhance the visibility of vegetation and differentiate it from other objects in the image. Vegetation indices are used to detect, quantify and monitor crop-related parameters such as percentage of vegetation cover and vegetation productivity. Distance to water in kilometres estimated the average distance from water sources to livestock grazing areas. Food insecurity measured the prevalence of moderate to severe food insecurity as defined by the Food Insecurity Experience Scale (FIES) that was developed by the Food and Agriculture Organization of the United Nations. It is an estimate of the percentage of a country’s population that faces difficulties in accessing enough safe and nutritious food for normal growth and development and an active and healthy life. The data were descriptively analysed using the Statistical Package for Social Sciences version 29. Data on climate were correlated to mortality and morbidity trends in children in Isiolo County using Spearman’s correlation.

### Qualitative data

#### Study population, sample size and sampling

The study population included two groups of key informants: parents or caregivers of children who accessed primary care and healthcare workers who worked with these children, including nurses, clinical officers and medical officers. The parents or caregivers had to have a child under 5 years, be residents of Isiolo County, and the child should have been diagnosed with a climate-sensitive disease between 2019 and 2023 at one of the primary care facilities. They needed to be adults (18 years or older), and the child should not have needed emergency medical care at the time of the interview. Healthcare workers needed to be employed by the Department of Health, Isiolo County and offer primary care to children under 5 years.

Purposive criterion sampling was used to select the study participants. Because of the multi-ethnic background of the region, the parents or caregivers interviewed were selected from the main tribes (the Somali, Meru and Borana people), with four people from each ethnic group. In addition, equal numbers of parents were selected from the outpatient clinic and inpatient wards. Parents were then selected consecutively according to their order in the queue or ward. All relevant healthcare workers were included. The sample size included 12 parents and six healthcare workers. The final sample size depended on data saturation, where no new potential themes were generated from the last two interviews with the parents.

#### Data collection

Qualitative data were collected from the key informants using semi-structured interviews. The interview guide was based on relevant literature,^18,25,26^ in line with the study objectives. Main topics in the interview guide were to explore the relationship of climate change to common climate-sensitive diseases. The framework in Figure 1 conceptualised this relationship.

Interviews explored the proximate causes that people experienced (e.g. food production and insecurity, access to fresh water), the mediating factors that may have increased or decreased vulnerability (e.g. poverty, culture, access to health care, service delivery) and the pathway to health and social effects (e.g. illness, migration). For parents, these issues were explored in the narrative of their child’s illness. For healthcare workers, these issues were explored from their experience of service delivery and the health system.

The interviews were audio-recorded and conducted in either of the national languages, which are English or Kiswahili, by well-trained research assistants. The study was piloted within Isiolo County Referral Hospital on two key informants, one caregiver and one health care professional, prior to the start of the study.

#### Data analysis

Qualitative data were transcribed verbatim and checked for any errors. The data were thematically analysed by the researcher with the help of Atlas-ti software. The researcher could analyse in both English and Kiswahili. The framework method was followed as outlined below^27^:

- Familiarisation: The first author (Beatrice W. Muhu) listened to the recordings, studied all the transcripts and field notes in order to recognise key ideas that informed the creation of a coding index.
- Identifying a coding index: The emerging ideas and issues from step 1 were captured in a series of well-defined codes. Related codes were sorted into categories and a coding index was created.
- Indexing: All the transcripts were coded using the coding index from step 2. New codes were added if necessary.
- Charting: Codes were grouped into families and reports were created within Atlas-ti that brought all the data together in one place.
- Mapping and interpretation: The reports were interpreted to identify themes and subthemes as well as any relationship between themes. Illustrative quotations were identified.

#### Trustworthiness

The researcher was a medical doctor who trained at the University of Nairobi and was registered as a medical practitioner by the Kenya Medical Practitioners and Dentists Council. She had practised medicine in different health facilities in Isiolo County. She had been involved in the primary care of children within the country and therefore had interacted with children who had been impacted by climate-sensitive diseases over the last few years. She had interacted with the parents of these children within the health facility as she was taking medical histories and managing various diseases related to climate change. She had also been part of the team of healthcare workers within the health facility responsible for service delivery. The researcher was conscious of her own thoughts and biases, having been part of the health system; however, all efforts were made to ensure openness and objectivity during the interview process. The research study was supervised by two qualified supervisors from Stellenbosch University, for example, the construction of the interview guide, piloting of the interviews, creation of the coding index and interpretation of the data. The researcher was assisted by three research assistants who were healthcare workers working in Isiolo County Referral Hospital. They were also part of the team responsible for primary care delivery among children; therefore, they were able to interact freely with the parents and healthcare workers as well as probe deeper into sensitive issues. The two data sources (parents and healthcare workers) were also triangulated in the analysis to give a more credible interpretation.

### Ethical considerations

Ethical clearance to conduct this study was obtained from Stellenbosch University Health Research Ethics Committee (No. S23/08/183).

## Results

The quantitative and qualitative findings are integrated into a series of themes according to the conceptual framework: (1) health and social effects, (2) mediating factors, and (3) proximate causes. The characteristics of the 18 participants in the qualitative interviews are presented in Table 2 and Table 3. Twelve participants were women between the ages of 22 years and 30 years with children under 5 years, and six were healthcare workers. The healthcare workers included nurses, clinical officers and a doctor with 3 years to 23 years of experience.

**TABLE 2:** Characteristics of the participants interviewed – Parents or caregivers.

Age (years)	Gender	Occupation	Age of child	Gender of child
29	Female	Housewife	3 years	Male
22	Female	Charcoal seller	5 years	Female
25	Female	Pastoralist	4 years	Male
29	Female	Self employed	4 years	Male
30	Female	Housewife	4 years	Female
22	Female	Businesswoman	4 years	Male
29	Male	Teacher	2 weeks	Male
24	Female	Teacher	7 months	Female
26	Female	Vegetable seller	3 years	Female
24	Female	Businesswoman	1.5 years	Female
24	Male	Pastoralist	2 years	Male
21	Female	Housewife	1 year	Male

**TABLE 3:** Characteristics of the participants interviewed – Healthcare workers.

Age (years)	Gender	Designation	Years of experience	Education level
25	Female	Clinical Officer	3	Diploma
55	Female	Nurse	23	Diploma
25	Male	Nurse	3	Diploma
30	Male	Paediatric Clinical Officer	6	University
46	Female	Nurse	15	Diploma
27	Male	Medical Officer	2	University

**FIGURE 3:**
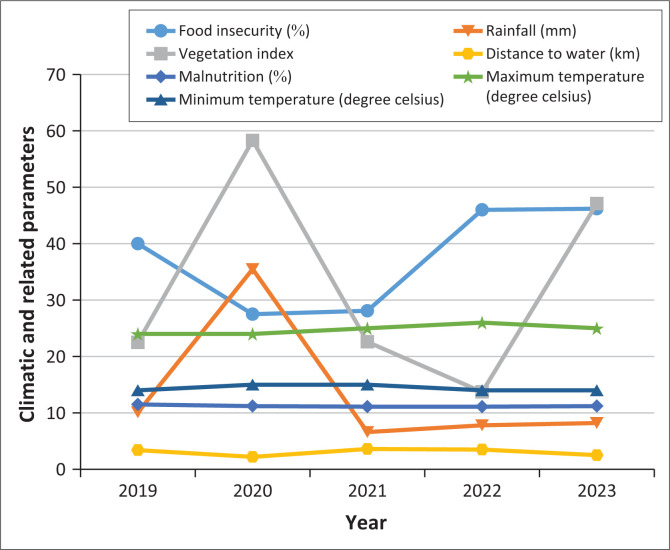
Trends in climatic and related parameters between 2019 and 2023 for Isiolo County, Kenya.

Table 4 presents the results on climate and related factors between 2019 and 2023, and Figure 3 depicts the trends graphically. Rainfall was highest in 2020 (35.3 mm) and lowest in 2021 (6.6 mm). The prevalence of food insecurity was highest in 2019 (40%) and lowest in 2020 (27.5%). Vegetation index was highest in 2020 (58.3%) and lowest in 2022 (13.7%). Distance to water sources was longest in 2021 at 3.6 km and shortest in 2020 at 2.2 km. Malnutrition levels were fairly constant across the years at 11% while mean temperatures showed little variation with maximum average temperatures at 26 °C and minimum average temperatures at 14 °C. The changes in food insecurity, vegetation index and distance to water sources were statistically significant, but not in rainfall, malnutrition and average temperatures. The higher the average rainfall per year, the higher the vegetable index, the lower the prevalence of food insecurity and the lower the distance to water sources. In 2023, there was a sporadic increase in rainfall in April and November, leading to flash floods. This led to an increase in vegetation index and a reduction in distance to water sources. Over this same period, the drought early warning system was reported at an alarm level for 44% of the months, alert for 19%, normal for 32% and in recovery for 3.4% with the highest alarms in 2021 and 2022.

**TABLE 4:** Table showing the distribution of climatic conditions and related parameters between 2019 and 2023.

Parameter	2019	2020	2021	2022	2023	*p*-value
**Food insecurity (%)**	-	-	-	-	-	< 0.001*
Median	40.0	27.5	28.1	46.0	46.2	-
95% Lower CL	33.5	25.0	25.3	40.2	42.9	-
95% Upper CL	47.4	30.4	40.3	49.3	56.1	-
**Rainfall**	-	-	-	-	-	0.982
Median	10.1	35.5	6.6	7.8	8.2	-
95% Lower CL	0	0	1.3	2.4	0	-
95% Upper CL	61.8	45.2	38.5	19.4	56.8	-
**Vegetation index**	-	-	-	-	-	< 0.001*
Median	22.5	58.3	22.6	13.7	47.1	-
95% Lower CL	18.0	49.7	18.7	11.2	13.6	-
95% Upper CL	24.0	76.6	27.9	17.1	59.5	-
**Distance to water (km)**	-	-	-	-	-	< 0.002*
Median	3.4	2.2	3.6	3.5	2.5	-
95% Lower CL	2.1	1.5	2.4	3.2	2.0	-
95% Upper CL	4.2	2.5	3.9	4.0	3.3	-
**Malnutrition (%)**	-	-	-	-	-	0.727
Median	11.5	11.2	11.1	11.1	11.2	-
95% Lower CL	9.5	11.2	8.5	9.9	9.7	-
95% Upper CL	15.6	16.7	13.9	13.2	14.2	-
**Max Temp °C**	-	-	-	-	-	0.107
Mean	24	24	25	26	25	-
95% Lower CL	23	23	24	25	24	-
95% Upper CL	25	25	26	28	26	-
**Min Temp °C**	-	-	-	-	-	0.395
Mean	14	15	15	14	14	-
95% Lower CL	13	14	14	13	14	-
95% Upper CL	15	16	15	15	15	-

Min, minimum; Temp, temperature; CL, confidence level.

* , *p* > 0.05 which is statistically significant.

### Proximate causes

The parents noted that temperatures had been on the rise over the last several years. While the average temperatures recorded in the data showed little variation, there were days or weeks with very high temperatures. This was seen to interfere with food security as many of the animals and plants did not thrive. As a result, malnutrition was perceived to be increasing. The healthcare workers observed that effects such as hyperthermia and dehydration were also prevalent during the dry season:

‘Once the drought starts, animals start to die or few of them survive. Due to the hot and dry climate, food is scarce.’ (29-year-old male, parent, teacher)

The residents explained that Isiolo was affected by flash floods mainly in April 2023 and November 2023. This destroyed farms, crops and domestic animals, which were the primary source of livelihood for many of the inhabitants. Business premises, traditional houses called ‘manyattas’ were swept away, causing homelessness and loss of income:

‘When the rain returned, it was accompanied by floods which swept away our homes and killed our animals.’ (29-year-old female, parent, housewife)

Parents commented that the drought significantly reduced their access to water. This reduction in rainfall was mainly observed between 2021 and 2023. Many of them mentioned that they relied on rivers and streams for water for their daily use. However, the water was noted to be inadequate and contaminated by various pollutants. Low rainfall also affected food production as crops had lower yields and animals frequently lacked pasture and water to drink:

‘Right now, there is little pasture for our animals and water for us to use. Plants and vegetation are also getting little rainfall causing them to wither and die due to the excessive heat.’ (26-year-old female, parent, businesswoman)

### Mediating factors

High out-of-pocket costs were emphasised as limiting access to healthcare. Despite public hospitals being affordable, a nurse reported that many families struggled with poverty and were not able to buy medication or seek laboratory services. Health insurance was seen to be inaccessible other than to those formally employed:

‘So, poverty is a big challenge here because of in terms of acquiring resources and treatment, they prefer our public facility because it is a bit cheaper. But in cases where we don’t have resources or our lab doesn’t have the common investigation that are required for managing children condition, you find that we are going to refer them outside which in such a case will not going to do those procedures that have been requested because they don’t have money.’ (25-year-old male, nurse)

Climate change was strongly observed to have adversely affected the residents’ main source of income, which was pastoralism. Other sources of income, such as kiosks, transport services and farming were also reported to be affected because of flooding, drought and famine. Some caregivers also commented that their employment was jeopardised because of absenteeism from taking care of sick children. This resulted in the redirection of funds from other priorities, such as food and education, to cater for medical costs:

‘Often, we go without food to get money to pay hospital bills.’ (22-year-old female, parent, charcoal seller)

During the floods, it was noticed that health facilities and medical equipment were destroyed. Infrastructure, such as roads and bridges, was also swept away with only a few vehicles that could transport people to hospitals. A nurse felt that ambulance services were not able to access sick children in remote areas, which frequently led to premature deaths:

‘The floods will sweep away roads so, the access to the facility will be very poor. In other places like those in the rural areas where there are dispensaries, flooding will take place in dispensaries.’ (27-year-old male, medical officer)

When outbreaks of illnesses occurred, it was observed that high admission rates led to overcrowding in the hospitals. This led to depletion of medical resources, healthcare worker shortages, burnout or mental health issues among medical staff, and extended stays of patients in the wards because of nosocomial infections. At the same time, attendance at maternal and child health (MCH) clinics for immunisations and wellness checks declined, as mothers were unable to physically or financially seek healthcare. Some of the healthcare workers noted that MCH attendance and vaccine coverage were particularly low during adverse weather events. Non Governmental Organisation (NGOs) and Faith Based Organisation (FBOs) were frequently relied on to supply medical care to remote areas during these times as a supplement to government-provided services:

‘The flow of clients is high and our staff they are very few, so, there is no enough staff to cater for the children who are brought to MCH department. Also, we have scarcity of resources. We don’t have enough drugs and commodities to maybe give or administer to our patients.’ (30-year-old male, paediatric clinical officer)

It was noted that caregivers were aware of some effects of climate change on health. However, many of them still had limited knowledge on this intersection. Language barriers were a major obstacle to awareness as many of the members of the community do not speak any of the national languages. Others felt that the lack of technological devices, such as smartphones hindered them from acquiring information on climate and health. Local administration, religious leaders and healthcare workers were often relied on to fill this gap within the community:

‘Those interior areas, the people who are living there, they don’t have access to the current technology. Maybe they don’t have TVs or radios where they can listen to the information that is being shared.’ (24-year-old male, parent, pastoralist)

### Health and social effects

Table 5 presents correlations between parameters and childhood illnesses. Spearman’s correlations indicated significant associations between food insecurity and both distance to water (*r* = 0.26, *p* = 0.048) and vegetation index (*r* = –0.52, *p* < 0.001). The greater the distance to water sources, the higher the prevalence of food insecurity. The higher the vegetation index, the lower the prevalence of food insecurity. Spearman’s correlations indicated no significant associations between climate change parameters and childhood conditions (*p* > 0.05) because of insufficient data.

**TABLE 5:** Correlations between childhood diseases and climatic and related parameters.

Category	Variables	Correlationcoefficient (r)	*p*-value
Correlations with food security	Distance to water (km)	0.260	0.048*
	Vegetation index	−0.522	< 0.001*
	Moderate acute malnutrition	0.300	0.600
	Severe acute malnutrition	0.600	0.300
	Pneumonia	0.200	0.800
	Malaria	0.800	0.100
Correlations with acute malnutrition	Vegetation index (%)	−0.158	0.236
	Rainfall	−0.010	0.942
	Maximum temperature	−0.229	0.084
	Vegetation index	−0.100	0.900
Correlations with malaria	Rainfall	0.300	0.600
	Distance to water	−0.400	0.500
	Food insecurity	0.800	0.100
Correlations with other diseases	Distance to water and diarrhoea	0.200	0.800
	Food insecurity and pneumonia	0.200	0.800

* , *p* > 0.05 which is statistically significant.

There were several diseases reportedly associated with changing climatic conditions: Waterborne diseases such as cholera or diarrhoea and vector-borne diseases such as malaria or kala-azar were perceived to affect young children because of several factors such as stagnant water pools, flooding, rising temperatures and contaminated water. Infectious diseases spread by close contact, such as measles, chickenpox and mumps, were thought to spread within schools or through interactions in the community. Preventive measures, such as mosquito nets, vaccines, clean drinking water and appropriate shelter, were often limited:

‘The common child illnesses, example malaria, are because people who are living far away don’t use nets and when it is raining there is an increase of mosquitoes. And things like cholera are common because people who are living at home don’t use the toilet, they just defecate anywhere. Another thing is measles. There is an increase of measles because they don’t come to clinics for vaccination.’ (Female Nurse, 23 years’ experience)

Emphasis was put on food scarcity within the region. Many parents felt that because of famine, there was inadequate food to give to their children. Many of them agreed that because of the high cost of living, prioritising a balanced diet for their families was difficult, as most of the food was frequently bought, not grown. Healthcare workers perceived that malnutrition, micronutrient deficiency and stunted growth were among the most common illnesses experienced in the region. Fruits and vegetables were noted to be often missing from the children’s diet as many of the pastoralist communities relied on meat, milk and blood for food:

‘During famine, there is food shortage to cater to the needs of all your children. Money to buy food is also limited so you can only afford one or two meals a day.’ (25-year-old male, parent, pastoralist)

It was noticed by the parents that many of them had poor access to clean, uncontaminated water as they used the same water for various uses such as drinking, laundry and washing dishes, among others. They also reported that they lacked pit latrines or lavatories, and because of their nomadic lifestyle, relieved themselves near rivers or water pools. Some also noticed that industries, hotels and several institutions emitted their effluent into the same water sources. Healthcare workers saw a rise in cholera, dehydration and diarrhoea during the flooding season as most of the water consumed was not treated:

‘Water is unavailable near our homes. We have to travel long distances to the river to fetch water which is often contaminated since we use it to drink, wash clothes and feed our animals.’ (26-year-old female, parent, vegetable seller)

It was mentioned that the dust, extreme temperatures and poor air quality might have contributed to pneumonia and upper respiratory tract infections. Although very few caregivers thought their children had frequent asthma exacerbations, they reported that many had allergic reactions to the cold or dust. Moreover, some caregivers felt there was an association between floods, cold temperatures and pneumonia because of the frequency of infections during these climatic conditions:

‘We also had respiratory conditions like pneumonia because of the cold. During the dry season due to the increase of dust, we had such condition like asthma due to the risk factors of allergies.’ (26-year-old male, nurse)

Caregivers commented that stress, anxiety and depression were experienced among family members as a result of their children’s illnesses, prolonged admissions and the financial implications related to healthcare:

‘We can also have emotional, stress, psychological trauma because the illnesses they build a lot of pressure to the family members. Like the guardians who take care of the children especially those who are admitted, find that they only go to the hospital leave the rest of the family unattended to, so may lead to psychological stress.’ (25-year-old male, clinical officer)

Displacement was expressed as a significant effect of the flooding in the region. Many families reported that they were rendered homeless and that they lost food, animals, property and businesses. It was also seen that access to hospitals and other social amenities was disrupted:

‘Flooding causes destruction of our homes forcing us to move to drier regions.’ (30-year-old female, parent, businesswoman)

A few caregivers reported that their communities struggled with insecurity and loss of animals because of banditry. This led to displacement, hunger and poverty as many people relied on animals for food and income:

‘You find that sometimes we fight, insecurity over food, over land, you find that people are displaced to go to other area whereby they don’t get the resources they are used to.’ (29-year-old female, parent, employed)

Caregivers noted with concern that some children were abandoned at home while their mother accompanied her sick child to the hospital. They often had to fend for their own food, take themselves to school and take care of themselves. Divorces were reported in a few families for various reasons, for example, if the father felt that the mother should have given more attention to her chronically ill child. It was seen that education was interrupted regularly because of either childhood illness or if finances were depleted from healthcare costs:

‘It affects the families, like now for example, when the mother concentrates on a sick child, it can bring out negligence. Maybe she is concentrating on this child and leaves the rest of the children at home. So, nobody will be taking care of those kids.’ (55-year-old female, nurse)

Some of the caregivers were initially reluctant to seek medical care in primary care facilities. Traditional healers and medicines were observed to be heavily relied on within the community. Prayers from religious leaders, such as Imams, were also reported to be utilised to treat ill children:

‘Most of these people are illiterate. They don’t believe in this medication. They believe in herbals and these herbal medications.’ (30-year-old male, paediatric clinical officer)

## Discussion

### Summary of the key findings

**FIGURE 4:**
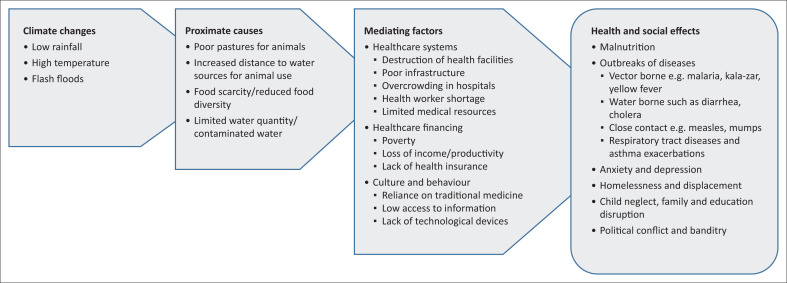
Summary of key findings.

Figure 4 shows a summary of the key findings organised according to the conceptual framework into climate changes, proximate causes, mediating factors, as well as health and social effects.

### Proximate causes

According to the Kenya National Drought Management Authority, Isiolo County experienced reduced rainfall in 2021 and 2022, which significantly reduced the vegetation index, and increased food security and the distance travelled to water sources. This led to a drought alarm being issued by the authorities.^28^ Similarly, the community’s experiences suggested that decreased rainfall had been observed throughout the region, leading to a predominantly hot and dry climate. In 2023, flash floods were experienced throughout the country, leading to the destruction of farms, animals and property. Temperature variation was not statistically significant within the study period.^23^ However, the community’s experiences suggested increased temperatures during that period. There was non-concordance between reports of heat waves from the respondents and regional meteorological data. This could be because of the differences between human lived experiences and actual temperature measurements. Other reasons could be the existence of local microclimatic changes in areas with loss of vegetation or shade, and perception bias experienced when local socio-economic activities, such as pastoralism, are influenced by heat stress.

### Mediating factors

Health expenditure continued to impoverish families due to the high cost of medication, laboratory investigations, hospital admissions, loss of income and productivity. This is because many parents had been forced to buy prescription medicines or laboratory investigations from private institutions because of public facility shortages. This was similar to findings from 49 other African countries that showed climate change had impacted out-of-pocket health expenditure.^29^ Social insurance had poor uptake within the communities because people prioritised their basic needs and could not afford to pay for insurance. This was similar to studies in Zambia, where only a small percentage of the population was willing to pay for social insurance.^30^

Climate shocks and stressors compounded the existing shortages of medical resources, healthcare workers and equipment, thus further reducing the quality of healthcare services. This was because of the destruction of health facilities and their resources. Accessibility of hospitals and dispensaries in remote areas was also reduced by the destruction of roads and bridges by floods. Emergency services were affected, resulting in premature deaths in children.^31^ Furthermore, existing resources were stretched by overcrowding in hospitals, with some children having extended stays because of low immunity and nosocomial infections. Healthcare workers were often overwhelmed, resulting in burnout and loss of productivity. As with other physicians, psychosocial factors, such as work demands, available resources and work environments contributed to their reduced well-being.^32^ Interestingly, immunisation coverage and attendance at MCH clinics declined during adverse climate events. This effect of climate change on vaccines has also been recognised by the Global Alliance Vaccine Initiative especially in low-income countries.^33^ Changes to vaccination planning had to be implemented to improve reach to remote areas.

The nexus of climate change and health was poorly understood by some members of the community. Similar to the general population in Egypt, where smartphones, TVs and radios were often used to gain knowledge, there was still a large gap in access to accurate and up-to-date information.^34^ A large part of the community depended on cultural practices and traditional medicine for the treatment of their sick children. Local administration, religious leaders and healthcare workers were often called on to give health talks, especially during disease outbreaks. Healthcare workers and other stakeholders require training programmes to build their capacity on climate change. Similarly, in India, there was proven to be a perceived gap among key stakeholders on the knowledge, vulnerabilities and public health risks of climate change, necessitating a need for awareness programmes and policies.^35,36^

### Health and social effects

The community experienced outbreaks of illnesses in their children, such as malaria, cholera and diarrhoeal diseases. Outbreaks of these diseases had not been experienced to this extent before and could be linked to the consequences of climate changes, such as flash floods. Outbreaks of diarrhoeal diseases such as cholera have also been observed in other low- and middle-income countries (LMICs), such as Malawi and Pakistan, following similar flooding.^37^ Malnutrition and micronutrient deficiencies were more common during the dry season. Unpredictable rainfall led to food scarcity in other regions in Kenya, such as Kilifi County, where the unavailability of food and lack of food variety resulted in poor nutritional status in children.^38^ Some of the parents reported their children were diagnosed with respiratory diseases, such as pneumonia and upper respiratory tract infections (URTIs). This was attributed to environmental influences and climate changes related to air quality and poor ventilation.^39^ Mental health disorders among children and their caregivers were noted because of deteriorating health in the children or the lack of adequate resources to take care of their families. Previous studies have also demonstrated that climate change has had an effect on child and adolescent mental health.^40^

Child neglect, threats of violence, family disruption, interruption of education and other social services for children were reported by some parents. This is similar to studies that have looked into the relationship between child abuse and climate change.^41^ Some of the parents commented that flash floods had left a trail of destruction to homes, businesses, farms and animals. Unfortunately, some people lost their lives during the floods in the region. Many other LMICs, such as India, China, Indonesia, the Philippines and Bangladesh, have also suffered effects such as deaths, displacement, destruction, separation of communities, and increased poverty as a result of flash floods.^42^ Political instability, banditry and crime increased in the region because of the loss of animals. This interruption in pastoralist activities in the region resulted in negative economic impacts because production of meat and milk had been adversely affected. Just as with rice production in Nigeria, political tension in rice farming regions reduced productivity as farmers would be attacked or intimidated during conflicts, keeping them away from their farming activities.^43^

### Strengths and limitations

Quantitative data on climate change in the region were adequate; however, there was a significant lack of data on childhood diseases from the local health services and national databases. Data on climate parameters were available in months, but data on climate-sensitive diseases were available in years. This made it difficult to analyse trends and correlations between climate changes and climate-sensitive diseases. The qualitative data came from users of the government hospital. People using the private or mission hospitals may have had different views or experiences. Those dependent on the government hospital may come from lower socio-economic groups and be more engaged in the informal sector for employment. Transferability of the findings to all people in this region may therefore be limited. Reflexivity may have been affected by culture as many of the participants came from conservative ethnic backgrounds, which may have influenced their openness during the interviews.

### Implications

Improving healthcare financing by providing affordable healthcare insurance as well as equipping public health facilities with resources would significantly reduce the financial burden on families. Increasing resilience in the healthcare systems by planning for adverse climates or investing in infrastructure could mitigate the impacts of climate on healthcare services. Closing gaps in information on climate and health among both healthcare workers and the community could be achieved by leveraging technology to disseminate knowledge. Strengthening data on climate-sensitive diseases from both local and national health information systems could significantly impact further quality research and development of effective policies based on community healthcare needs. Behaviour change can be promoted by engaging local leaders and communities to promote culturally acceptable programmes that would encourage modern healthcare practices. The Kenyan Ministry of Health has linked several adverse health outcomes, such as infectious disease risk and malnutrition, to climate stressors in the arid and semi-arid regions of the Country. To reduce these outcomes, there have been calls to enhance targeted interventions in health facilities to improve health system climate resilience and preparedness. Efforts to address health and social effects should focus on collaborations that will ensure food and water security. Addressing mental health challenges, homelessness, displacement and reduction of abuse among vulnerable populations, mainly children, can be integrated into adaptation policies.

## Conclusion

Climate changes have been experienced within Isiolo County over the last 5 years, mainly characterised by drought and high temperatures, but also compounded by sporadic high rainfall and flooding. This has led to food insecurity and changes in water availability and quality. The associations between climate change and climate-sensitive diseases among children were not made because of the paucity of data as well as the multifactorial nature of influences on health outcomes. The ability of children to cope with these challenges depended on their family’s income and financial situation, as well as the resilience and quality of primary care services. Primary care services were often of low quality even before the challenges of climate change. In many cases, climate change led to significant health effects such as malnutrition, malaria, diarrhoeal diseases and cholera. Substantial social effects included displacement, mental problems, child neglect and family disruptions. Efforts at adaptation should focus on poverty alleviation, the resilience of health services and facilities, as well as the ability to respond to the health and social effects.
